# A state hospital survey of movement disorders including intention tremor

**DOI:** 10.1192/bjo.2021.633

**Published:** 2021-06-18

**Authors:** Nigel Bark, Sung-Ai Kim, George Eapen

**Affiliations:** 1 Albert Einstein College of Medicine, Bronx Psychiatric Center; 2 Bronx Psychiatric Center (Retired); 3 Rainbow Babies and Childrens Hospital

## Abstract

**Aims:**

In a survey of movement disorders in patients in a State Hospital the finger-nose test was included because of increasing interest in the cerebellum in schizophrenia. It was expected that this would reflect the pathobiology of schizophrenia and be unrelated to the type of medication.

**Background:**

Abnormalities of movement and involuntary movements have gone from being considered part of schizophrenia to side-effects of medication to now demonstrably present in those who have never taken anti-psychotic medication. Soft neurological signs (SNS) are increased in schizophrenia, unrelated to medication, considered not to indicate brain localization, yet often include the finger-nose test which localizes to the cerebellum.

**Method:**

All available patients in a State Hospital were examined for movement disorders. They were rated on the following scales: Abnormal Involuntary Movement Scale (AIMS) for Tardive Dyskinesia (TD), Simpson-Angus Neurological Rating Scale for Parkinsonism (SANRS), Barnes Akathisia Scale (BAS), a Dystonia scale and the finger-nose test.

**Result:**

250 patients were included, 174 were examined or observed for movement disorder: 120 had no missing data, 54 refused part of the exam. Their mean age was 47, 62% male, 53% black, 26% Hispanic, 17% white.

Medication: First Generation Antipsychotic (FGA) 35 (mean CPZ equivalent dose:1177mg), Second Generation Antipsychotic (SGA) 159 (734mg), both FGA and SGA 56 (1907mg), no antipsychotic 3; anticholinergic or amantidine: FGA 57%, SGA 16%, both FGA and SGA: 50%.

Tardive Dyskinesia: all 23%, FGA 36%, SGA 25%, both 7%

Parkinsonism: all 38%, FGA 43%, SGA 33%, both 34%

Akathisia: all 3%, FGA 0%, SGA 4%, both 3%

Pseudo-akathisia: FGA 11%, SGA 4%, both13%

Dystonia: all 10%, FGA 13%, SGA 11%, both 8%

Intention Tremor: all 16%, FGA 0%, SGA 21%, both 16%

Half of those with Intention Tremor had Parkinsonism, a third had TD and a half were on anti-Parkinson medication.

None of these differences were statistically significant at p = 0.05 though intention tremor did show a trend (p = 0.08). The difference between FGA and SGA only became significant when all movement disorders were added together with those on anticholinergics with no movement disorder.

When compared with rates in similar State Hospitals in the 1970s tardive dyskinesia was now half the rate and Parkinsonism about the same.

**Conclusion:**

Overall rates of movement disorder are not very different between FGA and SGA. The surprise was that intention tremor only occurred with SGAs. Why?

